# Adverse infant outcomes following low-risk pregnancies in England: a retrospective cohort study

**DOI:** 10.1186/s12884-023-05598-2

**Published:** 2023-05-09

**Authors:** Megan Riley, Dimitra Lambrelli, Sophie Graham, Ouzama Henry, Andrea Sutherland, Alexander Schmidt, Nicola Sawalhi-Leckenby, Robert Donaldson, Sonia K. Stoszek

**Affiliations:** 1 GSK, 14200 Shady Grove Rd, Rockville, MD 20850 USA; 2 Evidera, 201 Talgarth Rd, Hammersmith, W6 8BJ London UK; 3 Present affiliation: Moderna, Cambridge, MA USA; 4 Present affiliation: Bill & Melinda Gates Medical Research Institute, Cambridge, MA USA

**Keywords:** Maternal vaccination, Infant outcomes, Low-risk pregnancy, Background rate, Maternal immunization trial, Pharmacovigilance

## Abstract

**Background:**

There are limited data describing adverse infant outcomes in infants born to women with a low risk of complications during pregnancy, such as those who may be enrolled in maternal immunization trials. This retrospective study estimated incidence proportions of infant outcomes in different cohorts of liveborn infants in England between 2005 and 2017.

**Methods:**

The incidence proportions of 10 infant outcomes were calculated for liveborn infants from pregnancies represented in the Clinical Practice Research Datalink (CPRD) Mother-Baby Link (MBL) and linkage to Hospital Episode Statistics (HES). Three infant cohorts were designed: (1) the all pregnancies infants cohort (*N* = 185,119), (2) the all pregnancies with a gestational age (GA) ≥ 24 weeks infants cohort (*N* = 183,869), and (3) the low-risk pregnancies infants cohort (LR infants cohort, *N* = 121,871), which included pregnancies with a GA ≥ 24 weeks and no diagnosis of predefined high-risk medical conditions until 24 weeks GA.

**Results:**

The most common adverse infant outcome in the three infant cohorts was macrosomia (e.g., 1,085.9/10,000 live births in the LR infants cohort), followed by minor congenital anomalies (e.g., 800.6/10,000 in the LR infants cohort), very low/low birth weight (e.g., 400.6/10,000 in the LR infants cohort), and major congenital anomalies (e.g., 270.4/10,000 in the LR infants cohort). The incidence proportions for early-onset sepsis, very low/low birth weight, and minor and major congenital anomalies were lower in the LR infants than in the other cohorts (non-overlapping confidence intervals [CIs]). The incidence proportions of neonatal death, infant death, late-onset sepsis, macrosomia, small for GA, and large for GA were similar between cohorts (overlapping CIs).

**Conclusions:**

This study generated background rates of adverse infant outcomes from liveborn infants of all and low-risk pregnancies represented in the CPRD Pregnancy Register MBL and linkage to HES. The results indicate lower incidence proportions of several adverse infant outcomes in infants from low-risk pregnancies compared to all pregnancies, illustrating the importance of considering maternal risk factors. These background rates may facilitate the interpretation of safety data from maternal immunization trials and of pharmacovigilance data from maternal vaccines. They may also be of interest for other interventions studied in pregnant women.

**Supplementary Information:**

The online version contains supplementary material available at 10.1186/s12884-023-05598-2.

## Background

Maternal immunization has become a key public health strategy to reduce the burden of infectious diseases in pregnant women and their infants [1, 2]. Vaccine-induced maternal antibodies are transferred through the placenta and persist after birth to protect infants in their first months of life, before the completion of their primary vaccination series [1–3].

Clinical development programs for maternal vaccines require enrollment of pregnant women in large-scale Phase III maternal immunization clinical trials [2–4]. To aid in the interpretation of safety data from such trials, it is key to standardize safety assessments after immunization [5] and understand background rates of adverse infant outcomes in infants from a population of mothers such as those who will be enrolled in maternal immunization clinical trials [4]. Generally, pregnant women who are considered as having a low risk of complications are more likely to be enrolled in maternal immunization trials than women with high-risk pregnancies. However, despite an established link between certain maternal characteristics (e.g., age, gestational diabetes) and adverse infant outcomes (e.g., large for gestational age [GA], macrosomia, neonatal death) [6–9], there is a gap in understanding the incidence of adverse infant outcomes in infants born to women with low-risk pregnancies. To address this knowledge gap, several studies were initiated, including a prospective cohort study assessing the rates of neonatal events of interest in liveborn infants in low- and middle-income countries (NCT03614676). Here, we report the results of a retrospective observational cohort study using data from the United Kingdom (UK) Clinical Practice Research Datalink (CPRD) and linked databases.

The study aimed to estimate the incidence proportions of adverse infant outcomes in three cohorts of liveborn infants from pregnancies represented in the CPRD Pregnancy Register Mother-Baby Link (MBL) and linkage to the Hospital Episode Statistics (HES) data: a cohort including infants from all pregnancies (AP infants), a cohort including infants from all pregnancies with a GA ≥ 24 weeks (AP24 + infants) and a cohort of infants from low-risk pregnancies with a GA ≥ 24 weeks (LR infants).

**Fig. 1:**
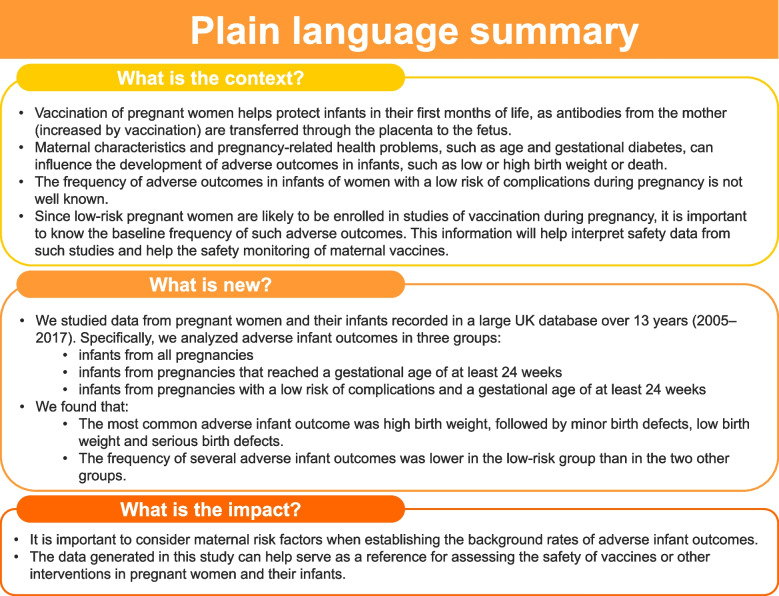
Plain language summary

The study also aimed to estimate the incidence proportions of pregnancy outcomes and pregnancy-related events of interest; these results are presented in an accompanying paper [10]. A plain language summary is provided in Fig. 1.

## Methods

The protocol of this study was approved by the Independent Scientific Advisory Committee for research involving CPRD data (protocol no. 18_144RA) and has been made available to the journal reviewers. Detailed methods are presented in the accompanying paper describing pregnancy outcomes and pregnancy-related events of interest from the same study [10]. An overview of the key elements of the study design and methodology are presented here.

### Study design and data sources

This was a retrospective observational cohort study which enrolled pregnancies and the infants that were born from these pregnancies registered in the CPRD, with data linked to the Pregnancy Register, HES, the Office for National Statistics (ONS), and the Index of Multiple Deprivation (IMD). For the analysis of adverse infant outcomes, additional linkage to MBL was required.

The CPRD Pregnancy Register uses a validated algorithm that identifies all pregnancy episodes in the CPRD GOLD database among women aged 11–49 years, with details about timing, duration, outcomes, and other features of the pregnancy [11, 12]. CPRD GOLD is one of CPRD’s two primary care databases and contains longitudinal de-identified patient record data (from 745 current and historic [of which 269 currently contributing] primary care practices across the UK at the time of data extraction in September 2018) and is considered representative of the UK population in terms of age, sex, and ethnicity [13, 14].

The MBL in the CPRD Pregnancy Register allows linking a mother’s medical records to those of her liveborn children [11]. The May 2018 version used in this study included > 1.3 million babies and 843,132 pregnancies between 1987 and 2018. While the Pregnancy Register captures all pregnancy outcomes, the MBL links mothers to infants for live births only. The MBL algorithm is based on data recorded in the primary care medical record and is updated monthly. At a per-pregnancy level, this identifies likely mother-baby pairs within the CPRD data, based on family identifier plus maternity information from the mother’s primary care record, and the month and/or year of birth of newly registered babies. The algorithm is designed to be specific (i.e., high confidence in the mother-baby pairs it identifies) rather than sensitive (i.e., it will not include all mothers and babies in CPRD GOLD).

HES provides diagnostic secondary care inpatient and outpatient records for England only [15] (thus restricting the analysis to pregnancies in England), the ONS provides information on the date and cause of all deaths recorded in England and Wales, and the IMD is a proxy for the socioeconomic status [15].

### Study period and population

**Fig. 2:**
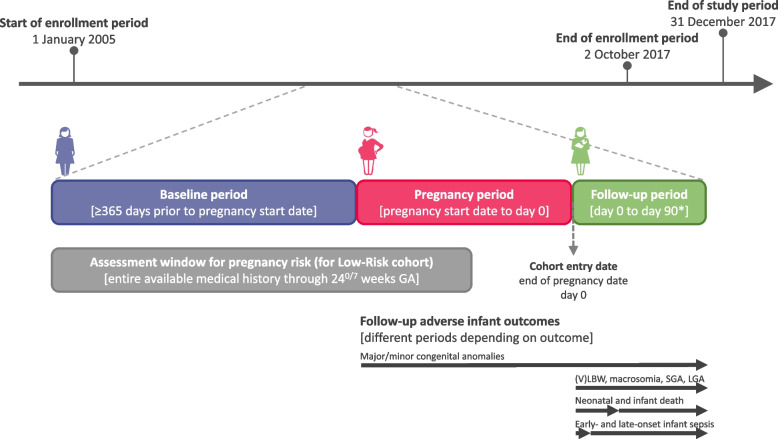
Overview of the study period. GA, gestational age; (V)LBW, very low/low birth weight; SGA, small for gestational age; LGA, large for gestational age. *A minimum of 90 days of active registration after the pregnancy end date was required for women/infants to be enrolled except if the woman/infant died during this 90-day period

Pregnancies in the CPRD Pregnancy Register with linkage to HES and with an end date from 1 January 2005 to 31 December 2017 were included if women were 18–45 years of age on the pregnancy end date and if they were continuously actively registered in the database from at least 365 days before pregnancy start (to be able to assess risk factors) until at least 90 days after pregnancy end (unless the woman died before the end of this period). To allow for this minimal 90-day follow-up after the pregnancy end date (to increase outcome ascertainment), only pregnancies with an end date up until 2 October 2017 were included (Fig. 2). Pregnancies associated with multiple births (e.g., twins, triplets) and with an unknown outcome were excluded. Exclusion criteria are reported in detail in the accompanying paper [10].

As described previously [10], three maternal cohorts were defined. The All Pregnancies cohort (AP cohort), including all eligible pregnancies, the All Pregnancies ≥ 24 weeks GA cohort (AP24 + cohort), including all pregnancies fulfilling the aforementioned criteria and excluding those with a GA record that did not reach 24^0/7^ weeks (calculated using the variable in the Pregnancy Register: “gestdays” < 168 days) and a Low-Risk cohort (LR cohort), including pregnancies from the AP24 + cohort without diagnosis of select high-risk medical conditions or procedures in the woman’s medical history (including all available medical history prior to the start of pregnancy through 24^0/7^ weeks GA) as detailed in the accompanying paper [10]. While the optimal window for administering a vaccine during pregnancy to maximize antibody transfer to the fetus has not been determined, the GA cut-off of 24 weeks reflects the cut-off chosen in previous group B streptococcus maternal immunization trials [16–18] and falls within the window during which maternal pertussis immunization is recommended in several countries [19].

**Fig. 3:**
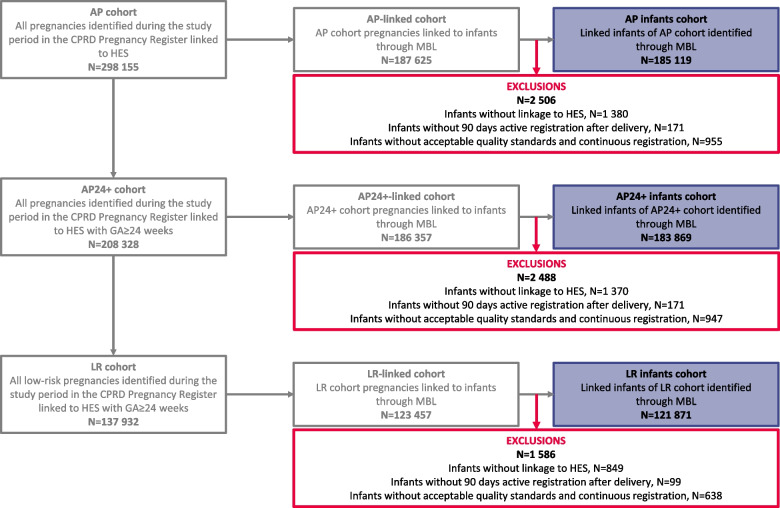
Cohort selection flow chart. AP cohort, All Pregnancies cohort; AP infants cohort; All Pregnancies infants cohort; AP24 + cohort, All Pregnancies with gestational age ≥ 24 weeks cohort; AP24 + infants cohort, All Pregnancies with gestational age ≥ 24 weeks infants cohort; LR cohort, Low-Risk pregnancies cohort; LR infants cohort, Low-Risk pregnancies infants cohort; CPRD, Clinical Practice Research Datalink; HES, Hospital Episode Statistics; MBL, Mother-Baby Link; GA, gestational age; N, number of pregnancies in the corresponding cohort/category

As not all pregnancies included in the Pregnancy Register can be linked to their infants using MBL, three additional pregnancy cohorts were generated: the AP-linked, the AP24 + -linked and the LR-linked cohorts, including pregnancies from the AP, AP24 + and LR cohorts, respectively, linked to their infants through the MBL (Fig. 3). Finally, three infant cohorts were defined: the AP infants, AP24 + infants and LR infants cohorts, including infants linked through MBL to pregnancies in the respective pregnancy linked cohorts. Inclusion in the infant cohorts was conditional on CPRD records being of acceptable quality and linkable to HES and infants being actively registered for at least 90 days after their date of birth (unless the infant died during this period).

### Study endpoints

Study endpoints were selected based on guidance from the Brighton Collaboration and the Global Alignment of Immunization Safety Assessment (GAIA) project on standardized case definitions for maternal immunization trials [5, 20]. However, these case definitions could not always be exactly applied because diagnostic laboratory results, procedure results, and medication prescribed during a hospital stay are underreported in the CPRD and linked datasets. Additionally, GAIA case definitions were not available for all study endpoints. Consequently, diagnostic codes were used (Read codes for CPRD and International Classification of Diseases, 10^th^ Revision [ICD-10] codes for HES) after manual alignment with GAIA definitions, where applicable (Additional file 1).

Adverse infant outcomes (listed in Table 1 with their assessment periods) were evaluated by inspecting both the mothers’ and infants’ records. All adverse infant outcomes, except neonatal and infant death, were identified based on Read codes in CPRD or ICD-10 codes in HES (and/or birth weight records in HES for very low/low birth weight and macrosomia). Neonatal death and infant death were assessed using dates of death from CPRD or ONS. If dates conflicted between both databases, the date from ONS was used. Information on how death was derived in the CPRD can be found in the accompanying paper [10]. The algorithms and codes used to identify the adverse infant outcomes can be found in Additional files 1 and 2, respectively. Information on the variables assessed in the study can be found in the accompanying paper [10].

**Table 1:** Infant events of interest

Infant events of interest
*Assessed during period as stated*
Neonatal death *(from birth to day 28)*
Infant death *(from day 29 to day 90)*
Early onset infant sepsis *(from day 0 to day 7)*
Late onset infant sepsis *(from day 8 to day 90)*
*Assessed from start of pregnancy until 90 days after birth*
Major congenital anomalies^a^
Minor congenital anomalies^a^
*Assessed from birth until 90 days after birth*
Very low (300–999 g) and low (1 000–2 499 g) birth weight^b^
Macrosomia (birth weight 4 001–7 000 g)^b^
Small for gestational age
Large for gestational age

^a^As the classification of major and minor congenital anomalies is only provided as International Classification of Diseases, 10^th^ Revision (ICD-10) codes in the European Surveillance of Congenital Anomalies (EUROCAT) [21], Read codes in the Clinical Practice Research Datalink were mapped manually to ICD-10 codes, and congenital anomalies were then classified as major or minor applying EUROCAT definitions, with major congenital anomalies being defined as: a structural or functional defect of prenatal origin, present at the time of live birth or fetal demise, or in utero, and affecting (or has the propensity to affect) the health, survival, or physical or cognitive functioning of the individual; and minor congenital anomalies being those with lesser medical, functional, or cosmetic consequences [22]. See Additional file 2 for the diagnostic codes

^b^Birth weight was set to missing if < 300 g or > 7,000 g

### Statistical analyses

Analyses were conducted using SAS software version 9.4 (SAS Institute Inc., Cary, NC, USA). No hypothesis testing was performed in this descriptive study. Potential differences between groups were based on non-overlapping 95% confidence intervals (CIs). Feasibility counts during protocol development indicated that the sample size obtained from the databases would provide sufficient precision for the descriptive purpose of the study. Standard data management practices were performed on the databases and missing values were identified but not imputed, assuming data were missing at random. To maintain confidentiality and individual data anonymization, data are shown only if at least five cases were observed for a given (sub)group.

Descriptive analyses for baseline demographic characteristics were conducted on the AP, AP24 + and LR cohorts, including number and proportion for categorical variables, and mean, standard deviation, median, interquartile range, and minimum and maximum values for continuous variables.

Within each infant cohort, the incidence proportion for each study endpoint was calculated as follows:

$$\frac{\text{Number of new cases of each adverse infant outcome in the period of interest}}{\text{Number of live births identified in CPRD in the period of interest}}\frac{}{}$$

Incidence proportions were calculated for every 10,000 live births, and 95% CIs were estimated using a generalized estimating equation model. This was done to minimize potential bias originating from including sequential pregnancies from the same woman as separate events in the cohorts, as such pregnancies are not independent [10].

Each study endpoint was presented for the entire study period and by calendar year of pregnancy start date. To calculate incidence proportions for the latter, outcomes were included in the calendar year in which the pregnancy started, which did not necessarily match the year in which the outcome occurred (e.g., if the adverse infant outcome occurred in 2013 and the pregnancy started in 2012). This was done to ensure that outcomes aligned with the denominator populations as these were based on pregnancies by start date per year.

## Results

### Sample selection and cohort description

The selection of pregnancies in the AP, AP24 + and LR cohorts is detailed in the accompanying paper [10]. Of the 298,155 pregnancies included in the AP cohort, 187,625 (62.9%) could be linked to their infants, and 185,119 infants were included in the AP infants cohort (Fig. 3). Of the 208,328 pregnancies in the AP24 + cohort, 186,357 (89.5%) could be linked to their infants, and 183,869 infants were included in the AP24 + infants cohort (i.e., 99.3% of the AP infants cohort) (Fig. 3). Of the 137,932 pregnancies in the LR cohort, 123,457 (89.5%) could be linked to their infants, and 121,871 infants were included in the LR infants cohort (Fig. 3). Reasons for exclusion from the different infant cohorts are depicted in Fig. 3.

### Demographic characteristics

Details on baseline characteristics of the AP, AP24 + and LR cohorts are described in the accompanying paper [10]. In brief, demographic characteristics were similar amongst these three pregnancy cohorts. The mean and median age of women at the start of pregnancy was 30 years and most women (≥ 84.0% of each cohort) were white.

### Adverse infant outcomes

#### Incidence proportions over the entire study period

The most common adverse infant outcome in the three infant cohorts was macrosomia (1,085.9 per 10,000 live births in the LR infants cohort and a little over 1,060/10,000 infants in the two other cohorts), followed by minor congenital anomalies (800.6/10,000 in the LR infants cohort, approximately 834/10,000 in the other cohorts), very low/low birth weight (400.6/10,000 in the LR infants cohort, approximately 453/10,000 in the other cohorts) and major congenital anomalies (270.4/10,000 in the LR infants cohort, approximately 298/10,000 in the other cohorts) (Table 2). The least common adverse infant outcomes were neonatal and infant death (1.4–1.8/10,000 and 1.8–2.4/10,000, respectively, in the three cohorts) (Table 2).

**Table 2:** Incidence proportions of adverse infant outcomes by study cohort

	**AP infants cohort *N* = 185 119**		**AP24 + infants cohort *N* = 183 869**		**LR infants cohort *N* = 121 871**	
	**n**	**Incidence/10 000 (95% CI)**	**n**	**Incidence/10 000 (95% CI)**	**n**	**Incidence/10 000 (95% CI)**
**Assessed during period as stated**						
**Neonatal death (birth–day 28)**	33	1.8 (1.3–2.4)	33	1.8 (1.3–2.4)	17	1.4 (0.9–2.1)
**Infant death (days 29–90)**	45	2.4 (1.8–3.2)	42	2.3 (1.7–3.0)	22	1.8 (1.2–2.6)
**Early-onset sepsis (birth–day 7)**	2 306	124.6 (119.5–129.8)	2 288	124.4 (119.3–129.7)	1 329	109.0 (103.2–115.2)
**Late-onset sepsis (days 8–90)**	818	44.2 (41.2–47.3)	809	44.0 (41.0–47.2)	501	41.1 (37.6–44.9)
**Assessed from birth to day 90**						
**Very low/low birth weight**	8 380	452.7 (442.9–462.6)	8 334	453.3 (443.5–463.2)	4 882	400.6 (389.3–412.1)
**Macrosomia**	19 718	1 065.2 (1 050.8–1 079.7)	19 543	1 062.9 (1 048.4–1 077.4)	13 234	1 085.9 (1 068.1–1 103.9)
**Small for gestational age**	918	49.6 (46.4–53.0)	915	49.8 (46.5–53.2)	565	46.4 (42.6–50.4)
**Large for gestational age**	1 910	103.2 (98.6–107.9)	1 896	103.1 (98.5–107.9)	1 185	97.2 (91.8–102.9)
**Assessed from start of pregnancy to day 90**						
**Minor congenital anomalies**	15 447	834.4 (821.8–847.3)	15 346	834.6 (821.9–847.5)	9 757	800.6 (785.3–816.1)
**Major congenital anomalies**	5 519	298.1 (290.4–306.0)	5 476	297.8 (290.0–305.8)	3 296	270.4 (261.4–279.8)

Incidence, incidence proportion per 10 000 live births; AP infants cohort, All Pregnancies infants cohort; AP24 + infants cohort, All Pregnancies with gestational age ≥ 24 weeks infants cohort; LR infants cohort, Low-Risk pregnancies infants cohort; N, number of linked infants included in the analysis in each cohort; n, number of linked infants in the specified category; CI, confidence interval

If a particular outcome occurred several times for the same linked infant, it was only counted once for that linked infant. More than one linked infant can be included for the same mother

As the AP infants and AP24 + infants cohorts comprised nearly identical populations, the incidence proportions in those two cohorts were comparable for all adverse infant outcomes (Table 2). The incidence proportions for early-onset sepsis, very low/low birth weight, and minor and major congenital anomalies were lower in the LR infants than in the other two cohorts (based on non-overlapping CIs, Table 2). Incidence proportions for the other outcomes (neonatal death, infant death, late-onset sepsis, macrosomia, small for GA, and large for GA) were similar across the three cohorts (based on overlapping CIs) (Table 2).

#### Incidence proportions by year of pregnancy start

**Fig. 4:**
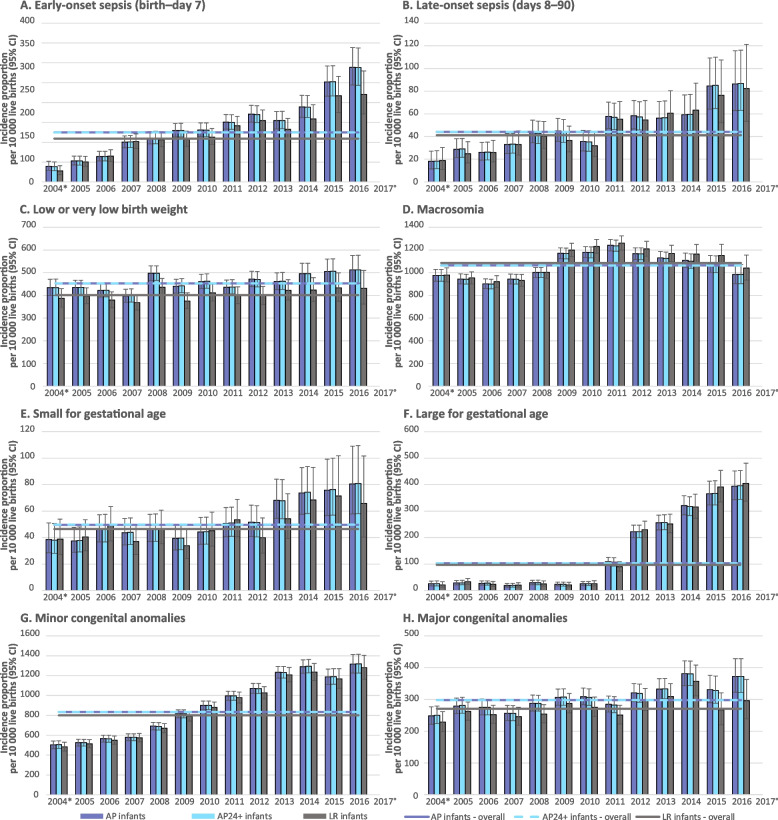
Incidence proportions of adverse infant outcomes by study cohort and calendar year of pregnancy start CI, confidence interval (depicted as error bars); AP infants, All Pregnancies infants; AP24+ infants, All Pregnancies with gestational age ≥24 weeks infants; LR infants, Low-Risk pregnancies infants *Because the study start date was 1 January 2005, pregnancies reported as starting in 2004 include only those which began in the last 9 months of 2004 (if full term, for example) °Pregnancies with a start date of 2017 were not included in this figure because the number was extremely low (and therefore incidence proportions less robust). Pregnancies reported as starting in 2017 included only those which began in the first month of 2017 (if full term, for example) because the study period end date was 31 December 2017 (with pregnancy end date up until 2 October 2017, as there was a requirement for at least a 90-day follow up after the pregnancy end date)

The incidence proportions of early- and late-onset sepsis, infants diagnosed as large for GA, and minor congenital anomalies increased substantially throughout the study period in each cohort (Fig. 4). Increases were strongest for the diagnoses large for GA (over 12-fold in each cohort comparing 2016 to 2005, with rises starting abruptly as of 2011) and early-onset sepsis (over fourfold comparing 2016 to 2005, with gradual rises throughout the study period) (Fig. 4).

An increase in the incidence proportions of infants diagnosed as small for GA was also seen, as well as a temporary rise in the incidence of macrosomia between 2008 and 2011 (Fig. 4). The incidence proportions of very low/low birth weight and major congenital anomalies remained relatively stable during the study period (Fig. 4).

Time trends in the incidence proportions of neonatal and infant death could not be analyzed because the number of deaths per year was too low (< 5 deaths per cohort most years).

## Discussion

This descriptive, retrospective cohort study using CPRD and linked data showed that the most common adverse infant outcomes in liveborn infants from pregnancies in England starting between 2004 and 2017 were macrosomia, followed by minor congenital anomalies, very low/low birth weight, and major congenital anomalies. It should be noted that the congenital anomaly outcomes were likely underestimated as these were only assessed in pregnancies resulting in live births.

Although no statistical tests for differences were performed in this descriptive analysis, the incidence proportions for early-onset sepsis, very low/low birth weight, and minor and major congenital anomalies were lower in the LR infants cohort compared to the other two cohorts (based on non-overlapping CIs). The incidence proportions for the remaining outcomes were similar between cohorts (based on overlapping CIs). These results suggest that the maternal risk profile influences the likelihood of developing certain infant outcomes more strongly than others.

The high incidence proportion observed for macrosomia in this study is in line with available estimates from ONS, showing that more than 10% of newborns had a birth weight of 4,000 g or higher in England and Wales in 2016 [23]. The estimates for major congenital anomalies in the AP infants cohort in the current study were higher than estimates reported by Public Health England (PHE), which showed an incidence of 205/10,000 total births (95% CI: 200.2–210.0) in England in 2016 [24]. This difference may result from different populations used as denominator: PHE used all births, including both live and stillbirths, while this study (based on MBL data) used live births only. The European surveillance of congenital anomalies reported 23.9 cases of major congenital anomalies per 1,000 births (of which 80% were live births) in 2003–2007 [25]. The incidence of neonatal and infant death (deaths occurring in the first 28 days and from day 29 to day 90, respectively) in this study (1.8 and 2.4/10,000 in the AP infants cohort) was more than tenfold lower than previously reported estimates of infant mortality (deaths occurring during the first year of life) for England based on ONS data: Nath et al. reported infant mortality rates of 36.7/10,000 live births in 2016 [26]. This difference could in part be explained by the definitions used. However, Nath et al.’s estimates for early and late neonatal mortality (deaths from birth to day 6 and during days 7–27: 21.4 and 5.6/10,000, respectively) were still higher than the estimates in the current study [26]. A possible explanation for this is that the MBL algorithm only includes infants that survive long enough for the algorithm to identify matching mother-infant pairs using information in both the mother’s and infant’s medical records. If an infant died soon after birth, the infant might not have enough information in its record to be identified by the MBL algorithm. Such deaths would therefore not have been included in the cohorts in this study, resulting in a lower reported incidence.

An increase in incidence proportions by calendar year of pregnancy start date was observed in this study for several adverse infant outcomes, most prominently for the diagnosis large for GA, starting from 2011. This increase may have been driven in part by coding changes; specifically, the Read code “large for dates” was introduced in 2010. This hypothesis is supported by the fact that macrosomia did not increase during the study period, except for a mild temporary rise between 2008 and 2011. A true increase in the incidence of large for GA cannot be excluded, particularly since the incidence of gestational diabetes, a known risk factor for this outcome [7, 9], also increased during the study period [10].

Increases in incidence proportions were also observed for early- and late-onset sepsis and, to a lesser extent, small for GA. The rising incidence of sepsis in infants parallels that observed for maternal sepsis in this study [10] and could be the result of a true rise combined with increased awareness and changes in screening and testing practices during the study period [27]. The increase in incidence proportion of small for GA observed after 2012 is in line with the increase seen for intrauterine growth restriction/poor fetal growth in the pregnancy cohorts in this study [10] and may also have been driven by coding changes as the Read code “small for age” was introduced in 2008. These results highlight that caution should be exerted when interpreting changes in incidence over time observed in retrospective studies based on routinely collected data, as observed changes may reflect a combination of a true change in incidence and changes in coding practice, clinical practice, and diagnostic or screening guidelines. Moreover, future changes in coding may affect the outcomes measured in the present study.

Additional strengths and limitations of this study are detailed in the accompanying paper [10].

Despite its limitations, this study provides valuable information on background rates of adverse infant outcomes and contributes to the evidence that maternal risk factors influence the rates of these outcomes.

## Conclusions

This real-world analysis based on primary and secondary care data from England generated background rates of adverse infant outcomes in infants from all and low-risk pregnancies represented in the CPRD Pregnancy Register MBL and linkage to HES. Understanding these background rates is crucial to facilitate the interpretation of safety data from maternal immunization trials and the monitoring of pharmacovigilance data from maternal vaccines. In addition, they may also be of interest for other interventions studied in pregnant women. While the LR cohort approximates the population most likely to be enrolled in maternal immunization trials, the other two cohorts provide valuable evidence for trials enrolling women at a high and low risk of complications and may serve as a reference for administration of vaccines to the general population of pregnant women. The results of this study, illustrating a lower incidence of some adverse infant outcomes in infants from low-risk pregnancies compared to infants born to all pregnancies, demonstrate the importance of considering maternal risk factors when establishing background rates of adverse infant outcomes.

## Supplementary Information

**Additional file 1.** Endpoints with GAIA definitions and the feasibility of applying these using CPRD data.

**Additional file 2.** List of codes used to identify adverse infant outcomes.

## Data Availability

Study documents can be requested for further research from www.clinicalstudydatarequest.com. The CPRD and linked data used in this study cannot be shared directly with others due to contractual agreements. CPRD data may be requested from enquiries@cprd.com.

## Abbreviations

AP cohort: All pregnancies cohort
AP infants cohort: All pregnancies infants cohort
AP24 + cohort: All pregnancies with a gestational age ≥ 24 weeks cohort
AP24 + infants cohort: All Pregnancies with a gestational age ≥ 24 weeks infants cohort
CI: Confidence interval
CPRD: Clinical practice research datalink
GA: Gestational age
GAIA: Global alignment of immunization safety assessment
HES: Hospital episode statistics
ICD-10: International classification of diseases, 10^th^ Revision
IMD: Index of multiple deprivation
LR cohort: Low-risk pregnancies cohort
LR infants cohort: Low-Risk pregnancies infants cohort
MBL: Mother-baby link
ONS: Office for national statistics
PHE: Public health England
UK: United Kingdom

## References

[1] Marshall H, McMillan M, Andrews RM, Macartney K, Edwards K (2016). Vaccines in pregnancy: The dual benefit for pregnant women and infants. Hum Vaccin Immunother.

[2] Vojtek I, Dieussaert I, Doherty TM, Franck V, Hanssens L, Miller J (2018). Maternal immunization: where are we now and how to move forward?. Ann Med.

[3] Omer SB (2017). Maternal Immunization. N Engl J Med.

[4] Munoz FM (2018). Current Challenges and Achievements in Maternal Immunization Research. Front Immunol.

[5] Bonhoeffer J, Kochhar S, Hirschfeld S, Heath PT, Jones CE, Bauwens J (2016). Global alignment of immunization safety assessment in pregnancy - The GAIA project. Vaccine.

[6] Kenny LC, Lavender T, McNamee R, O'Neill SM, Mills T, Khashan AS (2013). Advanced maternal age and adverse pregnancy outcome: evidence from a large contemporary cohort. PLoS ONE.

[7] Kim SY, Kotelchuck M, Wilson HG, Diop H, Shapiro-Mendoza CK, England LJ (2015). Prevalence of adverse pregnancy outcomes, by maternal diabetes status at first and second deliveries, Massachusetts, 1998–2007. Prev Chronic Dis.

[8] Lean SC, Derricott H, Jones RL, Heazell AEP (2017). Advanced maternal age and adverse pregnancy outcomes: A systematic review and meta-analysis. PLoS ONE.

[9] Reece EA (2010). The fetal and maternal consequences of gestational diabetes mellitus. J Matern Fetal Neonatal Med.

[10] Riley M, Lambrelli D, Graham S, Henry O, Sutherland A, Schmidt A (2022). Facilitating safety evaluation in maternal immunization trials: a retrospective cohort study to assess pregnancy outcomes and events of interest in low-risk pregnancies in England. BMC Pregnancy Childbirth.

[11] Clinical Practice Research Datalink (CPRD). Pregnancy Register and Mother-baby link. https://cprd.com/linked-data#Mother-baby%20link%20and%20Pregnancy%20Register. Accessed 9 June 2021.

[12] Minassian C, Williams R, Meeraus WH, Smeeth L, Campbell OMR, Thomas SL (2019). Methods to generate and validate a pregnancy register in the UK clinical practice research datalink primary care database. Pharmacoepidemiol Drug Saf.

[13] Clinical Practice Research Datalink (CPRD). https://cprd.com/home. Accessed 9 June 2021.

[14] Herrett E, Gallagher AM, Bhaskaran K, Forbes H, Mathur R, van Staa T (2015). Data Resource Profile: Clinical Practice Research Datalink (CPRD). Int J Epidemiol.

[15] Clinical Practice Research Datalink (CPRD). CPRD linked data. https://www.cprd.com/linked-data. Accessed 9 June 2021.

[16] Donders GG, Halperin SA, Devlieger R, Baker S, Forte P, Wittke F (2016). Maternal immunization with an investigational trivalent group B streptococcal vaccine: A randomized controlled trial. Obstet Gynecol.

[17] Heyderman RS, Madhi SA, French N, Cutland C, Ngwira B, Kayambo D (2016). Group B streptococcus vaccination in pregnant women with or without HIV in Africa: a non-randomised phase 2, open-label, multicentre trial. Lancet Infect Dis.

[18] Swamy GK, Metz TD, Edwards KM, Soper DE, Beigi RH, Campbell JD (2020). Safety and immunogenicity of an investigational maternal trivalent group B streptococcus vaccine in pregnant women and their infants: Results from a randomized placebo-controlled phase II trial. Vaccine.

[19] Kandeil W, van den Ende C, Bunge EM, Jenkins VA, Ceregido MA, Guignard A (2020). A systematic review of the burden of pertussis disease in infants and the effectiveness of maternal immunization against pertussis. Expert Rev Vaccines.

[20] Jones CE, Munoz FM, Kochhar S, Vergnano S, Cutland CL, Steinhoff M (2016). Guidance for the collection of case report form variables to assess safety in clinical trials of vaccines in pregnancy. Vaccine.

[21] EUROCAT. EUROCAT Guide 1.4 and Reference Documents. https://eu-rd-platform.jrc.ec.europa.eu/eurocat/data-collection/guidelines-for-data-registration_en. Accessed 9 June 2021.

[22] DeSilva M, Munoz FM, McMillan M, Kawai AT, Marshall H, Macartney KK (2016). Congenital anomalies: Case definition and guidelines for data collection, analysis, and presentation of immunization safety data. Vaccine.

[23] Office for National Statistics. Birth characteristics, 2016 workbook, Table 5. Live births (numbers and percentages): birthweight and area of usual residence, 2016. https://www.ons.gov.uk/peoplepopulationandcommunity/birthsdeathsandmarriages/livebirths/datasets/birthcharacteristicsinenglandandwales. Accessed 9 June 2021.

[24] Public Health England. National Congenital Anomaly and Rare Disease Registration Service: Congenital anomaly statistics 2016. https://assets.publishing.service.gov.uk/government/uploads/system/uploads/attachment_data/file/751553/Congenital_anomaly_statistics_2016.pdf. Accessed 9 June 2021.

[25] Dolk H, Loane M, Garne E (2010). The prevalence of congenital anomalies in Europe. Adv Exp Med Biol.

[26] Nath S, Hardelid P, Zylbersztejn A (2021). Are infant mortality rates increasing in England? The effect of extreme prematurity and early neonatal deaths. J Public Health (Oxf).

[27] Watson G, Caldwell C, Kennea N (2016). Neonatal early onset sepsis: a reflection on the NICE guidance. Infant.

