# Supplementary material for: Adverse infant outcomes following low-risk pregnancies in England: a retrospective cohort study
Source: BMC Pregnancy Childbirth. 2023 May 9;23:330. doi: 10.1186/s12884-023-05598-2 (PMC10170847; doi:10.1186/s12884-023-05598-2)
Supplement: Supplementary file 1 — Additional file 1. Endpoints with GAIA definitions and the feasibility of applying these using CPRD data. [file 12884_2023_5598_MOESM1_ESM.docx]

**Additional file 1. Endpoints with GAIA definitions and the feasibility of applying these using CPRD data**

| **Adverse infant outcome** | **GAIA definition** | **Identification algorithm** | **Feasibility of identification in Pregnancy Register (incl. MBL) and CPRD-HES** |
| --- | --- | --- | --- |
| Neonatal death [1] (assessed between birth and 28 days after birth) | Liveborn infant with gestational age <22 weeks OR birth weight <500 g AND death of infant in first 28 days of life  Liveborn infant with gestational age ≥22 and <28 weeks OR birth weight ≥500 g but <1000 g AND death of infant in first 28 days of life  Live born infant with gestational age ≥28 and <37 weeks OR birth weight ≥1000 g but <2500 g AND death of infant in first 28 days of life  Live born infant with gestational age ≥37 weeks AND birth weight >2500 g OR documented intra-uterine growth retardation if ≤2500 g AND death of infant in first 28 days of life | Death recorded as date of death in CPRD or ONS* | Expected to be well recorded in the data |
| Infant death (assessed between 29 and 90 days after birth) | No GAIA definition | Death recorded as date of death in CPRD or ONS* | Expected to be well recorded in the data |
| Infant sepsis, classified as early (first 7 days) and late (8 to 90 days) [2] | **Level 1**  Recognized pathogen identified using a validated method and from a normally sterile site  **Level 2**  Not meeting level 1 of evidence  AND  3 or more criteria:  - Temperature ≥37.5°C or <35.5°C  - Tachycardia or new or more frequent episodes of bradycardia  - New or more frequent episodes of apnea or increased oxygen requirement or increased requirement for ventilatory support  - Lethargy or moving only when stimulated or hypotonia or irritability  - Difficulty in feeding or abdominal distention  - Pallor or poor perfusion or  hypotension  - Abnormal white cell count or I/T ratio >0.2  - Abnormal platelet count  - Increased inflammatory markers  - Metabolic acidosis as defined by a base excess  **Level 3**  Not level 1 or 2 of evidence  AND  2 or more criteria:  - Temperature ≥37.5°C or <35.5°C  - Tachypnea or severe chest indrawing or grunting or cyanosis  - Change in level of activity  - History of feeding difficulty  - History of convulsions | Read codes in CPRD or ICD-10 codes in HES* | Diagnostic codes, especially during the first 7 days, expected to be underreported, especially in CPRD:  a) infant might not be registered with the general practitioner yet  b) the time frames investigated are very short and any real-world delays in coding to be reflected in the time periods assessed |
| Very low birth weight [3] | Very low birth weight: <1,500 g | Read codes in CPRD or ICD-10 codes in HES or infant has a weight recorded in HES that is 300–999 g (i.e., extremely/very low birth weight)* | Birth weight expected to be poorly recorded in CPRD, and in HES, birth weight is not reported for ~50% of births. Available codes partly distinguish between extremely low birth weight (≤999 g) and other low birth weight (1,000–2,499 g), and for others no specific weight is specified. Therefore, it may be challenging to differentiate between various scales of low weight. |
| Low birth weight [3] | Low birth weight: <2,500 g | Read codes in CPRD or ICD-10 codes in HES or infant has a weight recorded in HES that is 1,000–2,499 g (low birth weight)* |  |
| Macrosomia [4] | No GAIA definition  Standardized definition: birth weight >4,000 g | Read codes in CPRD or ICD-10 codes in HES or infant has a weight recorded in HES that is 4,001–7,000 g* |  |
| Small or large for gestational age [5] | Weight below 10^th^ percentile for gestational age  AND  the following used in assessment of weight:  - newborn weighed within 24 hours of birth  - weight assessed using a calibrated electronic scale with 10 g resolution  AND  the following for assessment of gestational age:  - certain LMP or IUI or embryo transfer date AND confirmatory ultrasound in first trimester  OR  - first trimester ultrasound  Birth weight >90% of newborns of same gestational age in the same population (>4,000 g at term) | Read codes in CPRD or ICD-10 codes in HES* |  |
| Major or minor congenital anomalies [6] | Major congenital anomalies:  a structural or functional defect with the following three characteristics:  - of prenatal origin  - present at the time of live birth or fetal demise, or in utero  - affecting (or has the propensity to affect) the health, survival or physical or cognitive functioning of the individual | Read codes in CPRD or ICD-10 codes in HES* | Major congenital anomalies likely to be better recorded than minor, however, there are still reports of major being underreported. |

GAIA, Global Alignment of Immunization safety Assessment; CPRD, Clinical Practice Research Datalink; MBL, Mother-Baby Link; HES, Hospital Episode Statistics; ONS, Office for National Statistics; ICD-10, International Classification of Diseases 10^th^ Revision; I/T ratio, immature to total neutrophil ratio; LMP, last menstrual period; IUI, intrauterine insemination.

*See additional file 2 for codes.

**References**

1. Pathirana J, Muñoz FM, Abbing-Karahagopian V, Bhat N, Harris T, Kapoor A et al. Neonatal death: Case definition & guidelines for data collection, analysis, and presentation of immunization safety data. Vaccine. 2016;34(49):6027-37.

2. Vergnano S, Buttery J, Cailes B, Chandrasekaran R, Chiappini E, Clark E et al. Neonatal infections: Case definition and guidelines for data collection, analysis, and presentation of immunisation safety data. Vaccine. 2016;34(49):6038-46.

3. Cutland CL, Lackritz EM, Mallett-Moore T, Bardají A, Chandrasekaran R, Lahariya C et al. Low birth weight: Case definition & guidelines for data collection, analysis, and presentation of maternal immunization safety data. Vaccine. 2017;35(48 Pt A):6492-500.

4. Mohammadbeigi A, Farhadifar F, Soufi Zadeh N, Mohammadsalehi N, Rezaiee M, Aghaei M. Fetal macrosomia: risk factors, maternal, and perinatal outcome. Ann Med Health Sci Res. 2013;3(4):546-50.

5. Easter SR, Eckert LO, Boghossian N, Spencer R, Oteng-Ntim E, Ioannou C et al. Fetal growth restriction: Case definition & guidelines for data collection, analysis, and presentation of immunization safety data. Vaccine. 2017;35(48 Pt A):6546-54.

6. DeSilva M, Munoz FM, McMillan M, Kawai AT, Marshall H, Macartney KK et al. Congenital anomalies: Case definition and guidelines for data collection, analysis, and presentation of immunization safety data. Vaccine. 2016;34(49):6015-26.
